# The Effects of Storage on Quality and Nutritional Values of Ehrenberg’s Snapper Muscles (*Lutjanus Ehrenbergi*): Evaluation of Natural Antioxidants Effect on the Denaturation of Proteins

**DOI:** 10.3390/biom9090442

**Published:** 2019-09-02

**Authors:** Abdelaziz Elgamouz, Rana Alsaidi, Alaa Alsaidi, Mostafa Zahri, Ahmed Almehdi, Khalid Bajou

**Affiliations:** 1 Department of Chemistry, College of Sciences, Research Institute of Science and Engineering, University of Sharjah, Sharjah P.O. Box 27272, UAE; 2 Department of Mathematics, College of Sciences, University of Sharjah, Sharjah P.O. Box 27272, UAE; 3 Department of Applied Biology, College of Sciences, University of Sharjah, Sharjah P.O. Box 27272, UAE

**Keywords:** fish, protein denaturation, spices, overfishing, Ehrenberg’s Snapper

## Abstract

Protein denaturation in frozen minced fillets (*Ehrenberg’s Snapper*), stored at −25 °C was studied; 50.0 mg biomass/50g mince fillets treated with cinnamon, cumin, turmeric, garlic, ginger and 25.0 mg of vitamin C were used to slow protein denaturation. FT-IR stretching vibration of Amide-A (νNH) at 3300 cm^−1^; Amide-I stretching (νC=O) between 1600−1690 cm^−1^ and Amide-II stretching (νCN) and bending (δNH) between 1480 and 1575cm^−1^ were used as marker peaks. Garlic was the most significant (*p* ≤ 0.01) in controlling the rate of protein denaturation when νNH was used as a marker peak. DSC analysis showed that turmeric presented the highest effect on delaying the denaturation of sarcoplasmic proteins with a $\Delta H^{0}=73.7J/g\;$ followed by garlic-treated mince fillets $\left(\Delta H^{0}=70.1J/g\right)$. All spices used were efficient in stopping the denaturation of myosin with the highest $\Delta H^{0}=769.3\;J/g$ registered for cinnamon-treated mince fillets. Actin was less vulnerable to denaturation in comparison to myosin and sarcoplasmic proteins.

## 1. Introduction

Recently, overfishing has been depleting and exhausting the fish populations worldwide. According to new statistics, the world consumption of fish has doubled from 9.9 kg in the year 1960 to 20.2 kg per capita in recent years [1]. Statistics proved that commercial fishing is in decline worldwide, resulting in a stress on the fishing sector and substantial mishandling of fish resources. Governments and international organizations are working together to overturn this situation by putting in place policy instruments such as the Common Fisheries Policy (CFP) and Good Environmental Status (GEnS) [2]. To increase awareness about overfished species in the area, the World-Wide Fund for Nature (EWS-WWF) has produced a fish guide to help consumers make informed choices about fish they want to consume. Its main aim is raising awareness about fish suffering from heavy exploitation and encourage consumers to control their habits over preferred overfished species [3]. Ehrenberg’s Snapper is a recommended fish species widely available in the Arabian Gulf according to Choose Wisely, a consumer awareness campaign of the Sustainable Fisheries Project launched in April 2010 [3]. Although the yearly catch of Ehrenberg’s Snapper fish is high, there is low demand for it in the market, therefore, freezing is the only way to extend the life of this very important source of proteins.

Protein denaturation during freezing storage has been reported in the literature [4]. Muscle proteins are predominantly vulnerable to freezing compared to plant-derived proteins, and this is specifically true for fish species [5]. This is mainly due to the formation of ice crystals, dehydration, and concentration of solutes in the tissue or protein solution [6]. Myofibrillar proteins are the most vulnerable because they can be subjected to structural changes when interacting with assorted kinds of lipids or lipid oxidation products during frozen storage [7,8]. Lipid-rich fishes containing high levels of polyunsaturated fatty acids, that are susceptible to oxidation, resulting in off-flavours, changes in colour and texture and loss of nutrients [9]. Hydroperoxides are initial lipid peroxidation products which decompose due to instability, generating numerous secondary products, such as aldehydes that can subsides to food rancidity. Along with aldehydes’ cytotoxic activity attributed to their reactivity with nucleophiles and sulfhydryl groups of protein and nucleophilic acids or related amino acids. The mechanism of aldehyde toxicity was attributed to aldehydes being able to crosslink with different compounds including proteins in muscles causing stiffness to those muscles [10].

**Figure 1:**
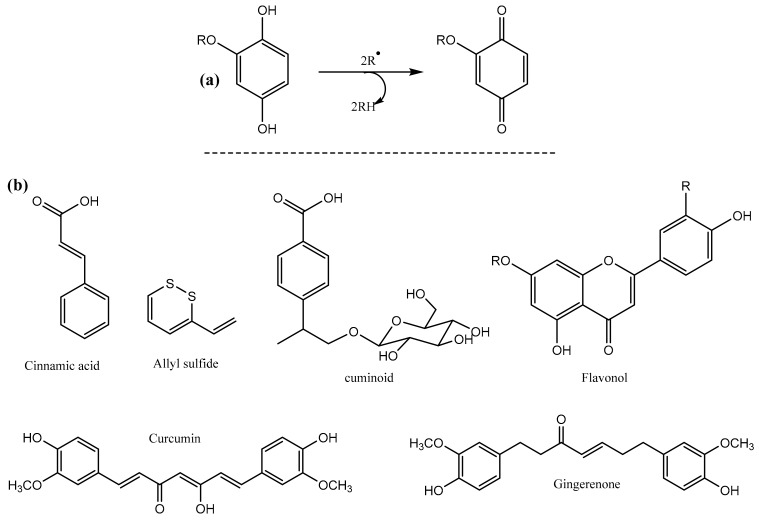
(**a**) Reaction of quinone mediator formation after interaction with radical species and (**b**) Structures of flavonoid and active phenolic compounds found in cinnamon, garlic, cumin, turmeric and ginger.

On the other hand, antioxidants act as inhibitor of protein denaturation by retarding compounds that interfere with one of the steps of chain propagation or initiation that lipids undergo during oxidation [11]. Phenolic antioxidants were found to be strong chemoprotectors against carcinogen-induced carcinoma due to their ability to scavenge reactive radical species [12]. Their protective effect is mediated by quinones formed as a result of first interaction with reactive oxygen species (Figure 1a). Some representative phenolic antioxidants found in the spices used in this study are given in Figure 1b.

The interaction of proteins and polyphenols has been widely studied in food products. Foods are generally regarded as a collection of nutrient and bioactive molecules that are essential to human health. Polyphenols and proteins may be assembled into aggregates due to the natural affinity of proteins for medium polarity of polyphenols. Mainly non-covalent bonding are responsible for stabilising such complexes [13], even though, covalent bonding may occur.

This paper examines the biochemical composition of Ehrenberg’s Snapper as an alternative for the overfished categories in the UAE such as Greasy grouper (*Epinephelus tauvina*) [3]. Also, examines the effect of antioxidants contained in extracts of widely used spices in the local kitchen, namely: cinnamon, cumin, turmeric, garlic, and ginger in inhibiting the denaturation process of proteins during storage at −25 °C. Vitamin C was used as a reference pure synthetic antioxidant. Changes in protein structure, thermodynamic parameters and the texture which occur during the storage process were assessed by FT-IR spectroscopy and differential scanning calorimetry (DSC). To understand the interaction between antioxidants and the proteins, the polyphenols and flavonoids contents as well as the DPPH scavenging activities of various spices were studied. Also, the biochemical composition of Ehrenberg’s snapper was studied in terms of lipids, proteins, proteins, moisture, ash and metal contents.

## 2. Materials and Methods

### 2.1. Materials

Methanol (CH_3_OH, HPLC grade 99.9%), petroleum ether (ACS reagent), 2,2-diphenyl-1-picrylhydrazyl (DPPH, C_18_H_12_N_5_O_6_, ≤100%), sodium hydroxide (NaOH; ≥98.0%), sodium nitrate (NaNO_2_; ≥ 97.0%), aluminium chloride (AlCl_3_; 98%), potassium iodide (KI; ≥99%), chloroform (CH_3_Cl; ≥99.5%), glacial acetic acid (CH_3_COOH; 100%), sodium thiosulfate (Na_2_S_2_O_4_; 99%), sodium carbonates (Na_2_CO_3_, 99%); soluble starch ((C_6_H_10_O_5_)_n_; ign. residue ≤0.4%), rutin (C_27_H_30_O_16_·xH_2_O, ≥94%; HPLC grade), (gallic acid; ≥ 99%; HPLC grade) potassium sulfate (K_2_SO_4_; ≥ 99.0%); copper sulphate (CuSO_4_.5H_2_O; ≥ 99.0%); titanium oxide (TiO_2_; ≥ 99.0%)_,_ boric acid (H_3_BO_3_, 99%), methyl orange (1% solution), Folin–Ciocalteu’s phenol reagent (2N) were purchased from LabcoLtd, the Local representative of Sigma-Aldrich. Vitamin C (C_6_H_8_O_6_; 100%) was purchased from a local pharmacy. Ehrenberg’s Snapper fish was purchased from Aljubail Seafood Market, Sharjah, UAE, they were delivered in ice to the working laboratory in a period of time less than 1 h from the purchase time. Ginger, turmeric, garlic, cinnamon and cumin were purchased from a local market.

### 2.2. Methods and Instrumentation

#### 2.2.1. Extraction of Spices Using Methanol and Water

Cinnamon sticks, dry ginger roots, dry turmeric roots, and cumin seeds were grinded separately using coffee grinder (Black&Decker, Beijing, China)). Fresh garlic was crushed using a pestle and mortar. 5.00 g of each spice weighed in 250 mL conical flask and extracted with 100 mL of water or methanol in a horizontal mechanical shaker for 2 h at temperature of 80 °C. After cooling down, the solutions were gravitationally filtered using a Whatman filter (Cambridge, England). The extracts were dried on a rotary evaporator (Bibby scientific, Stone, England) to remove the solvents; 150.0 mg of the dry spice was dissolved in 100 mL of water or methanol and transferred into amber sample bottles, to avoid interaction with the light, bottles were labelled and stored in the fridge for further uses; 50.0 mg of Vitamin C was dissolved in 100 mL of methanol or water, to get a concentration of 500 ppm. Vitamin C was used as a pure synthetic and reference antioxidant. Methanol extracts were used for polyphenols and flavonoids analysis while water extracts were used for the marinating of fish samples. Methanol extract were used to quantify the concentration of total polyphenols and flavonoids in different spices’ extracts due to the fact that reagents used in the test of phenolic and flavonoids are soluble in methanol but not in water.

#### 2.2.2. Total Phenolic Content (TPC)

The total phenolic contents of garlic, ginger, cumin, turmeric and cinnamon were determined by using the spectrophotometric method of Singleton and Rossi [14]. 1.00 mL of Folin–Ciocalteu’s phenol reagent was added to 1.00 mL of methanol containing 1.00 mg/mL of dry spice and mixed together. After 5 min, 10.0 mL of a 7.00 (*m*/*v*)% Na_2_CO_3_ solution was added to the mixture. The mixture was diluted by adding 13.0 mL of deionised water and was shaken using the rotamixer. The reaction mixture was kept in the dark at a temperature of 23.0 °C for 90 min then absorbances were measured at 750 nm using a spectrophotometer (UV–2510TS–Labomed Los Angeles, CA, USA). The same procedure was performed with gallic acid concentration ranging from 25.0 to 400 mg/L. Results were expressed in mg of gallic acid equivalent per 100 g of sample dry mass (mg (GAE)/100 g DW).

#### 2.2.3. Total Flavonoids Content

Total flavonoids contents of garlic, ginger, cumin, turmeric and cinnamon were measured according to the method of Park et al., [15]. In 10.0 mL test tubes, 0.30 mL of each methanolic spice extract stock solutions (0.5 mg/mL, 1.0 mg/mL and 10.0 mg/mL) was mixed separately with 3.40 mL of 30.0 (*v*/*v*)% methanol, 0.15 mL of 0.50 M NaNO_2_ and 0.15 mL of 0.30 M AlCl_3_.6H_2_O. After 5 min, 1.00 mL of 1.0 M NaOH was added to the mixture. A blank was prepared by mixing the same reagents without any spice extracts. A standard curve of rutin in the range of 10.0–80.0 ppm was prepared from 400 ppm stock solution. Sample solutions and standards were homogenized, then absorbances were measured at 356 nm using a UV–VIS Spectrophotometer (UV–2510TS–Labomed).

#### 2.2.4. DPPH Radical Scavenging Activity of the Polyphenols

Modified method of Brand-Williams et al., [16] was used to determine the DPPH scavenging activity of garlic, ginger, cumin, turmeric and cinnamon extracts; 2.00 mL of 0.50 mM DPPH freshly prepared in methanol were introduced to five test tubes containing 2.5 mL of each spice methanolic extract stock solution (1.5 mg/mL) and were incubated at room temperature for a period of 30 min to allow reaction to take place. The UV–Vis absorbances were measured at a wavenumber of 517 nm using a UV–VIS Spectrophotometer (UV–2510TS–Labomed). Methanol was used as a blank. Absorbances of stock solutions represent the control absorbance (A_before_) of the test and $A_{after}$ is the test’s absorbance. The DPPH radical scavenging activity was evaluated from Equation (1).

(1) $$DPPH\left(inhibition\right)=\frac{A_{before}-A_{after}}{A_{before}}\times 100$$

#### 2.2.5. Sample Preparation

A total of 2.0 kg of Ehrenberg’s Snapper was gutted, cleaned under running water and made into fillets. The fillets were minced using an electric mincer (Aftron, Beijing, China) to give 844 g. This mass was divided into four batches. The first batch made by weighing seven portions of 56.0 g of mince fillets which were marinated with 33.0 mL of water extract of the appropriate source of antioxidant, in a ratio of (1.0 mg of biomass/g of mince fillets). The other three batches, lipid analysis, protein analysis and reference were made by weighing 56.0 g of minced fillets for each batch in three polyethylene boxes, no spice was added to the three batches. All batches were kept overnight in the fridge, except lipids’ batch, to allow penetration of antioxidants into the mince fillets, then transferred to the freezer at −25.0 °C and kept for a period of one month, sampling was made every week.

#### 2.2.6. Lipid Extraction from Ehrenberg’s Snapper

A total of 10.0 g of freshly prepared mince fillets taken from the lipid analysis batch was placed into the thimble of Soxhlet apparatus (Quickfit, England). Petroleum ether (90.0 mL) was added to a round-bottomed flask as extracting solvent, boiling chips were added to reduce bubbles during the distillation process. The condenser was mounted on the tumble of Soxhlet apparatus. Then, the mixture was boiled for a period of 3 h at 70 °C. Petroleum ether was filtered and evaporated in the rotary evaporator. Lipid content were determined from Equqtion (2).

(2) $$Lipid\;Content\;=\frac{Lipid\;mass\;\left(g\right)\;}{mass\;of\;minced\;fish\;\left(g\right)}\times \;100$$

#### 2.2.7. Determination of Peroxide Value (PV)

Peroxide value was measured according to AOCS method [17]; 0.18 g of fish oil was weighed in a dry conical flask, then suspended in 10.0 mL of chloroform, 15.0 mL of glacial acetic acid and 1.00 mL of freshly prepared potassium iodide (KI). The conical flask was tightly closed and gently swirled to allow its contents to mix for 1 min and kept for another 1 min in the dark. 1.00 mL of starch solution (2.00% *m*/*v*) and 75.0 mL distilled water were added to the mixture. The solution was titrated with 0.01 M sodium thiosulfate (Na_2_S_2_O_3_). The indicator was added towards the end of the titration while the pale straw colour is still present. The solution was shaken during titration until the blue colour disappeared. A blank titration was carried out under the same conditions on a mixture containing all reagents used in the test except the oil. No more than 0.50 mL of 0.01 M sodium thiosulfate solution should be consumed for this purpose. The PV value was calculated from Equation (3).

(3) $$PV(\frac{meq}{Kg})=\frac{(V_{1}-V_{0})\times C\times 1000\times T}{m}\times \;100$$

In Equation (3), V_1_ represents the volume dispensed from 0.01 M Na_2_S_2_O_3_ in the main test. V_0_, is the volume dispensed from 0.01 M Na_2_S_2_O_3_ in the blank test. C, the molar concentration (molarity) of the Na_2_S_2_O_3_. T, is the titre of Na_2_S_2_O_3_ and m, the mass of fish mince fillets extracted with petroleum ether in grams.

#### 2.2.8. Determination of Moisture Content

Three glass watches were oven-dried at 100 °C for 30 min, cooled in a desiccator then their masses were recorded. 5.00 g of fish tissue was placed in each pre-weighed glass watch and kept in the oven at 100 °C for 24 h. The glass watch was removed from the oven and cooled in a desiccator (Duryea, PA, USA), the experiment was run in triplicates, masses were recorded and the moisture content was then calculated from Equation (4).

(4) $$\%\;\;Moisture\;=\frac{mass\;loss\;on\;drying\;at\;100\;{}^{\circ}C\;}{mass\;of\;sample}\times 100$$

#### 2.2.9. Ash and X-ray Fluorescence (XRF) Analysis

Samples were prepared by weighing 6.00 g of fish tissue into dry pre-weighed silica dishes in triplicate. Initially, the dishes with fish tissue were heated on a hot plate for ten minutes to remove the moisture. Followed by heating in a muffle oven at 550 °C for seven hours. After the formation of ash, the dishes were removed, cooled in a desiccator, and weighed. The ash content was determined using Equation (5).

(5) $$mass\;of\;ash\;\left(g\right)=mass\;of\;silica\;dish\;with\;ash\;-\;mass\;of\;silica\;dish$$

The composition in metals of fish ash was analysed by an XRF instrument (XGT-7200 X-ray Analytical Microscope; (Horiba, Irvine, CA, USA) to look for any toxic heavy metals.

#### 2.2.10. Measurement of Protein Content Using the Kjeldahl Method

Protein content of Ehrenberg’s Snapper was measured based on the organic nitrogen content via the Kjeldahl procedure [18]. Briefly, 1.00 g of the minced fillets was digested in 20 mL of (H_2_SO_4_, 96%) together with two selenium catalyst tablets (5.0 g K_2_SO_4_; 0.15 g CuSO_4_.5H_2_O; 0.15 g TiO_2_). The mixture was boiled in a distillation apparatus for 2 h. The digestion of the minced fillets continued until a clear solution was developed. Then the flask was left to cool down for 15 min. This technique is based on the conversion of nitrogen present in proteins to ammonia in the form of ammonium sulphate; 20.0 mL of 0.50 M NaOH was added to allow the release of ammonia via steam distillation in the distillation apparatus, and the distillate was collected over 25.0 mL of boric acid (4.00% *m*/*v*) then titrated against a standard solution of 0.05 M Na_2_CO_3_ using methyl orange as indicator. The nitrogen (%) and protein content (%P) were determined using Equations (6) and (7). Where K is a factor used for meat products, it is equal to 6.25.

(6) $$\%N\;=\frac{\left(ml\;titrant\;of\;sample-ml\;titrant\;blank\right)\times 0.0007}{weight\;of\;sample}\times \;100$$

(7) % P = N×K 

#### 2.2.11. Fourier Transform Infrared Spectroscopy

FT-IR spectra were recorded using an FT-IR Bruker Platinum Spectrometer (Bruker, Hamburg, Germany) fitted with an attenuated total reflection (ATR) unit, with single reflection geometry. Small amount of frozen sample was allowed to melt at room temperature then introduced into the ATR unit. Intensities of fish samples spectra treated with different natural source of antioxidant were recorded three times at room temperature in the transmittance mode using the average of 16 scans to produce a single spectrum. All spectra were collected at a resolution of 4 cm^−1^ across a range of 4000 to 500 cm^−1^. Analysis of samples were made on a weekly basis. Different spectra are given in Figures S2 to S5. Peaks of interest were, NH stretching vibration of amid A at 3300 cm^−1^, C=O stretching vibration of Amide-I between 1600−1690 cm^−1^, CN stretching and NH bending vibrations of Amide-II between 1480−1575 cm^−1^. A table of peaks of interest for each spice treated and non-treated mince fillets was made by recording transmittance of samples at 1 week, 2 weeks, 3 weeks and 4 weeks storage timing. Data is given in Table S5 in Supporting Documents. The data was computed using SPPS as described in the statistical analysis.

#### 2.2.12. Differential Scanning Calorimetry

The denaturation process during freezing of the samples along with the reference sample was further studied by using Differential Scanning Calorimetry (TA instruments, New Castle, DE, USA), A power compensation device DSC-Q20 instrument equipped with TA RCS 90 refrigerating system and TA Instruments Universal analysis 2000 v.4 software was used to compare T_m_ and ΔH^0^ of the reference sample with Tm and ΔH^0^ of the samples treated with different sources of antioxidants after 30 days of storage at −25 °C. T_m_ is the temperature at which half of the sample is transformed and was measured at the tip of the peak. Analysis was made in aluminium sample pan (T151207) filled with roughly 8.0 mg of mince fillets and covered with a cover type (T15126), the sample pan and the cover were hermitically sealed. The reference container was filled with distilled water with the same mass as the sample and was adjusted within ± 0.1 mg of the same mass. Scanning was made between 20 and 120 °C at a heating rate of 5.0 °C/min under a nitrogen flow of 50.0 mL/min. The cooling back to 20 °C was made with the same rate of 5.0°/min. DSC analysis was made for the most vulnerable samples which are expected to present most denatured structures of proteins after 4 weeks.

### 2.3. Statistical Analysis

The results were calculated from the averages of all sample readings and represented as Mean ± SD. The treatment of data was performed using SPSS 15.0 version for windows. Descriptive analysis one-way ANOVA and pair-wise comparison of mean values of different variables by the *t*-test were used with a significant level of *p* < 0.05.

## 3. Results and Discussion

The total phenolic contents of the spices are represented in Table S1 in mg(GAE)/100 DW (Supporting Document). Table shows that (TPC) increase in the following order: 3.73 ± 0.01, 5.92 ± 0.02, 13.26 ± 0.01, 20.34 ± 0.01 and 37.93 ± 0.19 for garlic, ginger, cumin, turmeric and cinnamon respectively. Table S2 (in Supporting Document) shows the content in rutin for different spices, cumin methanolic extract has the greatest flavonoids content among the rest of the methanolic extract with an estimated value of 129.40 ± 0.05 ppm of rutin at high concentrations. While the methanolic extract of fresh garlic shows the lowest flavonoids content with an estimated value of 26.13 ± 0.01 ppm of rutin at high concentrations. The content in rutin was found to increase with the increase of the concentration of the biomass, the lowest value was registered for garlic at 0.58 ± 0.31 ppm.

DPPH scavenging activity has been widely used to test the ability of many compounds as free radical chelating scavengers, or even as hydrogen donors. In some cases, DPPH was used to evaluate the antioxidative activity of plant extracts [19,20]. Table S3 (in Supporting Document) gives the results of DPPH scavenging activity of different sources of antioxidants, it was shown that the antioxidant activity increases with the increase of biomass concentration. The activity varies between 0.27 ± 0.01 for garlic as lowest value at lowest concentration to 90.54 ± 0.01 for cumin at highest concentration. The decreasing order of DPPH scavenging activity at high concentration was found as follow: Ginger (90.543% ± 0.01) > cinnamon (90.50% ± 0.01) > cumin (89.873% ± 0.01) > turmeric (80.45% ± 0.01) > Vitamin-C (26.26% ± 0.01) > garlic (7.10% ± 0.01). The same order was followed at low concentrations. No trend could be established between the TPC or rutin contents in the spice and its DPPH scavenging activity; this is due to different mechanisms and compounds contained in each spice which can affect reaction rates, and consequently, the antioxidant activity results [21].

Lipid content of Ehrenberg’ s Snapper was found to be 4.20 ± 0.07%. Depending on their fat content, fish are classified as lean (up to 2.0% fat), medium fat (2–7% fat), fat (7–15% fat), and very fat (over 15% fat) [22]. Accordingly, Ehrenberg’s Snapper fish can be classified as medium fat fish. Marine oils including fish, mussels are highly susceptible to oxidation due to the substantial number of polyunsaturated fatty acids (PUFA) they contain. The peroxide value (PV) of Ehrenberg’s Snapper was found to be 9.94 ± 0.03 meq/kg of oil. Fish oil generally has very low peroxide values; as low as 1.0 meq/kg according to Di Giorgio et al., [23]. The value obtained in our test is indicative that mince extracted were not fresh and that lipid oxidation had already begun but still in an early stage. Values of more than 30.0 meq/kg have been reported by the same authors when fish oil is mixed with soy proteins with a 3:1 (fish oil/soy bean protein) ratio.

The moisture content of Ehrenberg’s Snapper by an oven drying method was found to be 73.98% ± 0.12. Ash content of the Ehrenberg’s Snapper was found to be 8.17% ± 0.44; these values are comparable to values obtained by Kaminski et al. when studying the ash content of larvae of barbel, *Barbus barbus* (L.) [24].

High contaminated fish products with heavy metals (As, Cd, Cu, Hg, Pb and Zn) were documented in the Arabian Gulf; specifically, Hg and As displayed the highest concentrations [25]. Data of elements’ concentrations in Ehrenberg’s Snapper ash are summarized in Table S4 (in Supporting Document). Results show the presence of one single heavy metal (Zn) with a concentration of 171.50 mg/kg, which is higher than 50.0 mg/kg, the accepted value by the FAO [26]. Other elements do not present any risk for human consumption.

The estimated value for nitrogen using the Kjeldahl method was found to be %N = 2.48% ± 0.25. By multiplying the %N by the factor used in meat products (K = 6.25), the percentage of proteins was estimated to be 13.66% ± 0.25. Overall, the chemical constituents of fish are water (60–84%), proteins (15–25%) and minerals (0.8–2%), small amounts of carbohydrates (glycogen) and vitamins (0.3%) [27]. The chemical composition of Ehrenberg Snapper, including moisture, total lipids, protein and ash found in this study are presented in Figure S1 (in Supporting Documents). Moisture content is the major component, constituting 73.98% ± 0.28 of the total mass, followed by proteins at 13.66% ± 0.25, total lipids at 4.20% ± 0.21 and ash at 8.16% ± 0.04 constituting a total of 99.10% the remaining 0.90% could be attributed to volatile compounds.

**Figure 2:**
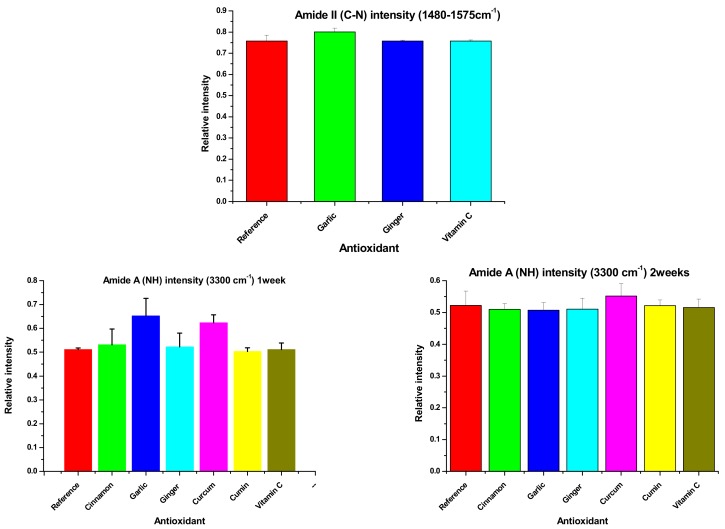
Amide-II (C-N) intensity (1480−1575cm^−1^) after 1week, Amide-A (NH) intensity (3300 cm^−1^) after 1week and 2weeks for antioxidant treated and non-treated minced fillets of Ehrenberg’s Snapper at −25.0 °C. Data are expressed as the mean ± SD, n = 3.

Figure 2 shows the Amide-A NH (3300 cm^−1^) FT-IR symmetrical stretching vibration (νNH). This vibration is insensitive to protein conformation; it depends frequently on the strength of the hydrogen bonding [28]. Denaturation and conformational changes in proteins are observed by the changes in the environment around the side chains of amino acids. No significant increase was observed in the intensities of νNH (*p* > 0.05) in all samples with the use of different sources of antioxidants at the same time of storage, except for turmeric. Turmeric-treated Ehrenberg’s Snapper’s mince fillets present the highest increase (mean difference value = 0.1122 ± 0.0270), showing that turmeric has the lowest effect on inhibiting the protein denaturation. Comparison of antioxidant effects reveals that a significant increase was observed in the intensities when comparing garlic with other sources of antioxidant as well as turmeric with other sources of antioxidant (*p* < 0.05). The highest difference was found between garlic and turmeric (mean difference value = 0.1492 ± 0.0590) followed by garlic and vitamin C (mean difference = 0.1411 ± 0.0470) then between garlic and ginger (mean difference = 0.1298 ± 0.0170).

After two weeks of storage time, a significant difference was observed in the (ν_NH_), only between the reference and turmeric (mean difference value = 0.0291 ± 0.0110), indicating that turmeric has the lowest effect on inhibiting protein denaturation. When comparing antioxidants effects to each other, a significant difference could be observed between all other sources of antioxidants and turmeric. This supports the first result that turmeric has the lowest effect on protein denaturation inhibition. While the second significant difference was observed between vitamin C and garlic, showing that garlic has the second lowest effect after turmeric (mean difference value = 0.0086 ± 0.0011).

The intensities of the Amide-A ν_NH_ were followed up for two additional weeks (weeks 3 and 4). No significant changes were found (*p* > 0.05) between the reference and antioxidant-treated fillets or between antioxidant-treated fillets themselves; this may find an explanation in the fact that after two weeks the NH stretching vibration is affected by hydrogen bonding formation [29]. Ice crystals will be induced by extending the freezing time to 3 and 4weeks which led to reduction in the intensity of NH amide vibration [5].

Significant differences were observed in the amide-II C-N stretching vibration (ν_CN_) at 1480 cm^−1^, between the reference and the three sources of antioxidant; garlic, ginger and vitamin C, with most significant change observed between vitamin C and the reference (mean difference 0.0610 ± 0.0160), showing that vitamin C has the highest effect on inhibiting protein denaturation followed by ginger and garlic with mean differences of (0.0340 ± 0.0113) and (0.0420 ± 0.0147) respectively.

**Figure 3:**
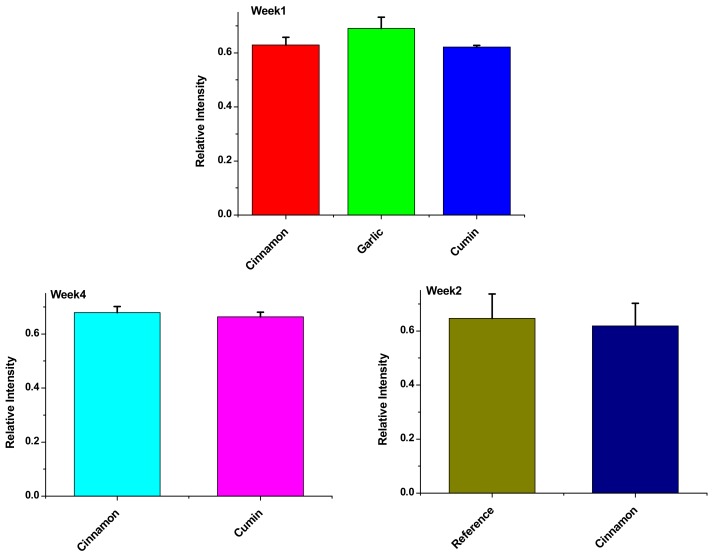
Amide-I (C=O) (1600−1690 cm^−1^) for antioxidant treated and non-treated minced fillets of Ehrenberg’s Snapper at −25.0 °C at different timing from 1week to 4 weeks. Data are expressed as the mean ± SD, n = 3.

Figure 3 represents Amide-I C=O stretching vibration (ν_CO_) at 1600 cm^−1^, significant differences were observed from week1 (*p* < 0.05) between garlic and cumin and between cumin and cinnamon; the highest change was observed between cinnamon and cumin (mean difference 0.00815 ± 0.0033) indicating that cumin has the highest effect in inhibiting protein denaturation. On increasing the storage time from two weeks to four weeks, a significant difference could be observed in week 2 between the reference and cinnamon, but after 4 weeks of storage time, cumin remains with the highest effect on delaying protein denaturation with a mean difference of 0.0211 ± 0.0058.

**Figure 4:**
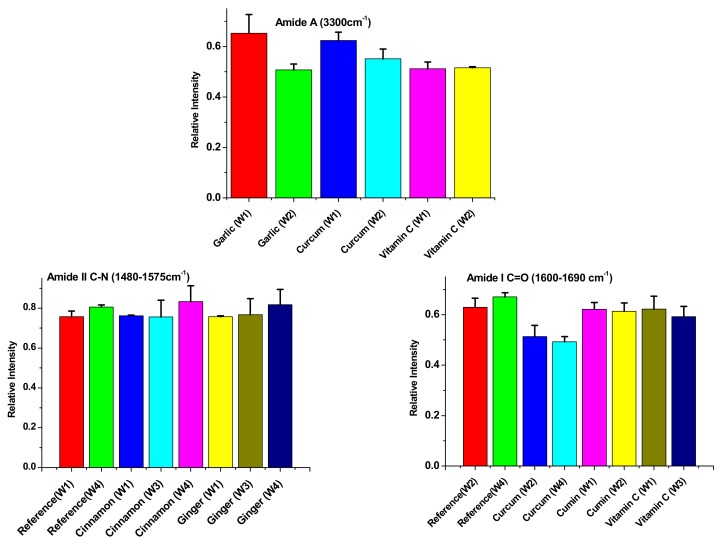
Amide-A intensity (3300 cm^−1^); Amide II C-N (1430–1676 cm^−1^) and Amide I C=O (1600−1690 cm^−1^) for antioxidant treated and non-treated minced fillets of Ehrenberg’s Snapper at −25.0 °C at different timing from 1week to 4 weeks. Data are expressed as the mean ± SD, n = 3.

Figure 4 represents the effect of storage time from 1 week to 4 weeks on the three main assessed FT-IR vibrations (ν_NH_, ν_CO_ and ν_CN_). For Amide-A ν_NH_, the most significant change was found between garlic at week 1 and garlic at week 2 with a mean significant difference of 0.1456 ± 0.0500, showing that garlic has the highest effect on stopping protein denaturation. Turmeric was shown to have the second significant difference between week 1 and week 3 (mean difference = 0.0722 ± 0.0060) and the least significant change was found for vitamin C between week 1 and week 2 (mean difference = 0.0182 ± 0.00300). While, for ν_CO,_ the most significant change was found between reference at week 2 and week 4 (mean difference = 0.0821 ± 0.0243); turmeric shows the second and third significant changes between weeks 2 and 4 and between weeks 1 and 2 with mean differences of 0.0382 ± 0.0135 and 0.0352 ± 0.0071, respectively. Also, vitamin C shows significant changes between week 1 and week 3. As far as ν_CN_ is concerned, the most significant changes were manifested by ginger between weeks 2 and 4 followed by weeks 1 and 2, with a mean differences of 0.1832 ± 0.0454 and 0.1594 ± 0.0176, respectively, followed by two consecutive changes of cinnamon between weeks 1 and 2 and between weeks 2 and 4 with mean differences of 0.0469 ± 0.0148 and 0.1142 ± 0.0482, respectively. The lowest change was observed for the reference between weeks 1 and 4 with a mean difference of 0.0666 ± 0.0272.

Overall, the FTIR study shows that garlic is the most efficient source of antioxidants as far as the NH stretching vibration at 3300 cm^−1^ is concerned, while ginger was found to be efficient when using the CN stretching vibration as a marker peak. Fresh garlic was found to have the lowest scavenging DPPH activity (7.10% ± 0.01 for 10,000 µg DPPH/mL of antioxidant) and also the lowest concentration in antioxidants, 26.13 ± 0.01 mg rutin/L and 3.73 ± 0.01 mg(GAE)/100 DW. This shows that the concentration of the antioxidant does not matter but what matters is the nature of antioxidant and the site of interaction for both antioxidant or protein. Undesirable results may be obtained sometimes during the interaction of the protein and antioxidant [29].

Changes in thermodynamic parameters for frozen Ehrenberg’s Snapper mince fillets in the presence and absence of different sources of antioxidants; 50.0 mg garlic; 50.0 mg cinnamon; 50.0 mg cumin; 50.0 mg turmeric; 50.0 mg garlic; 50.0 mg ginger; and 25 ppm vitamin C per 50.0 mg of mince fillets; and without antioxidant for control after 30 days of storage at −25.0 °C are represented in Figures S6 to S12 (Supporting Documents), peaks (°C) and enthalpies (J/g) obtained for different antioxidants treated mince fillets from DSC are summarized in Table 1.

In DSC analysis, melting point temperature (T_m_) of fish muscles could be used to gain information about the optimum condition for the storage of fish [30]. Two proteins are of interest in this study; sarcoplasmic and myofibrillar proteins. It is known that fresh fish fillets present two main endothermic peaks at 54.7 °C and 75.0 °C in DCS, which correspond, respectively, to myosin and actin [31]. However, according to Hashimoto et al., [32], these peaks shift to high temperature when samples are moist. In this study, the peak at lower temperatures (42.0–49.0 °C) is attributed to myosin. The second important peak from 60 to 76.0 °C is attributed to sarcoplasmic proteins, and the third peak between 105 and 112 °C is attributed to actin. It is known also in DSC that, at melting temperature, half of the protein is folded, and the other half is unfolded, it is therefore, expected that the endothermic peaks of proteins under consideration were reduced as indication that they have been partially unfolded [33]. Equation (8), represents the equilibrium between these two thermodynamic states.

(8) $$F\;\overset{\begin{array}{c} Thermodynamic\;\\ equilibruim \end{array}}{\Leftrightarrow }\;U$$

In Equation (8), F represents the protein at folded state and U, its unfolded state. At this temperature, different thermodynamic parameters could be expressed by Equation (9) to Equation (13).

(9) $$K=\frac{U}{F}=1$$

(10) $$\Delta G^{0}=\Delta H^{0}-T_{m}\Delta S^{0}$$

(11) $$\Delta G^{0}=\Delta H^{0}-T_{m}\Delta S^{0}=0$$

(12) $$\Delta H^{0}=T_{m}\Delta S^{0}$$

(13) $$T_{m}=\frac{\Delta H^{0}}{\Delta S^{0}}$$

K represents, the stability constant, $\Delta H^{0},$ is the enthalpy, $\Delta S^{0},$ the entropy and $\Delta G^{0},$ is the Gibbs free energy of the protein denaturation process at its melting temperature, T_m_. At this stage, the protein is 50% folded and 50% unfolded and $\;\Delta G^{0}=0$.

Thermograms of untreated and antioxidant treated Snapper’s mince fillets show an endothermic peak between 35 to 49.0 °C corresponding to myosin. T_m_ changes for garlic and vitamin C treated mince fillets were higher than T_m_ value of the reference. However, T_m_ values for cumin and cinnamon-treated mince fillets were lower than the reference’s T_m_. This change was not observed in the case of ginger and turmeric. The second important endothermic peak displayed by all DSC thermograms of mince treated with different antioxidants and non-treated ones is the broad peak between 60.0 and 77.0 °C which corresponds to transitions due to the denaturation of sarcoplasmic proteins. All treated samples show high T_m_ values compared to the reference when comparing non-treated with cumin, garlic and turmeric-treated mince fillets with most significant increases at 77.0 and 76.5 °C found for cumin and turmeric, respectively. High temperatures can lead to the precipitation of the sarcoplasmic proteins and negatively affect the water holding the myofibril proteins such as myosin and actin and therefore, stimulate the denaturation in mince fillets [34]. The third important transition appears between 105.0 and 112.0 °C, and it is ascribed to actin melting. No significant changes were observed in this transition, showing that actin is more solid than previously assessed proteins, myosin and sarcoplasmic proteins [34]. An actin peak shows an increase in the case of turmeric, garlic, cumin and a decrease in the case of cinnamon, ginger and vitamin C. Overall, no trend could be observed for T_m_ values for myosin, sarcoplasmic and actin, T_m_ values are known to be dependent on the moisture content of the sample. The glass transition temperature was found to drop as the moisture content increases; T_m_ is expected to follow a similar trend according to Hashimoto [33]. It is therefore recommended to focus on the enthalpy changes rather than melting point when studying the protein denaturation using DSC.

All treated Snapper’s mince fillets showed high enthalpies compared to the reference as far as the myosin is concerned, this could be explained based on the fact that treatment of mince fillets with natural source antioxidant stabilizes the protein and more energy is needed to unfold it. High configurational entropies were also observed because when proteins are unfolded few stabilizing interactions could be adopted, there is an entropy enthalpy compensation because ΔG = ΔH − TΔS, increased temperatures favour the unfolding and therefore, the entropy term more heavily. The highest effect was observed for cinnamon treated mince fillets with a $\Delta H^{0}=769.3\;J/g\;$followed by cumin with a $\Delta H^{0}=\;91.2\;J/g$. Similar results were found by Badii and Howell [35], when studying the effect of vitamin C and vitamin E, as cryoprotectants, on the denaturation of frozen Cod (Gadus morhua), the enthalpies of the myosin treated were found to be higher than the myosin untreated samples. This was explained by stabilization of myosin by cryoprotectant molecules. No myosin peak was observed in the case of turmeric and ginger, this could find an explanation in that, ginger and turmeric contain cysteine proteases that may have degraded myosin more preferentially than actin. It is known that cysteine proteases, present in many plant extracts cleaves myosin at the base of the heads, releasing two myosin fragments and a long tail shaped like a rod [36].

The most significant increase in the enthalpies of sarcoplasmic proteins was observed when comparing Snapper non-treated mince fillets with turmeric-treated mince fillets $\left(\Delta H^{0}=73.7J/g\right)\;$followed by garlic-treated mince fillets $\left(\Delta H^{0}=70.1J/g\right),$ showing that turmeric has the highest effect in stopping the denaturation of the sarcoplasmic proteins. No sarcoplasmic peaks were assessed in the case of cinnamon and ginger-treated mince fillets. Heating the sarcoplasmic proteins leads to their coagulation and release of calcium cations (Ca^2+^), the increase of Ca^2+^ concentration has a direct effect on the ionic strength of the water holding the myofibril proteins and, therefore, induces denaturation by disrupting the salt bridges holding different structures of the proteins. Similar results were observed when studying the gel properties of Sardine (*Sardinella Gibbosa*) [37]; denaturised sarcoplasmic proteins bound to myofibrils were found to reduce the water-holding capacity (WHC) of sardine meat. WHC, were found also to be improved when pork meat was treated with sodium bicarbonate (NaHCO_3_), the improvement was attributed to the increase of the ionic strength provided by NaHCO_3_ [38]. Highly significant changes were observed in the enthalpies of all treated mince fillets with different antioxidants as far as actin is concerned. The most significant change was found for ginger-treated mince fillets $\left(\Delta H^{0}=1367J/g\right)$ followed by turmeric $\left(\Delta H^{0}=482.8\;J/g\right)$ showing that turmeric has the highest effect in delaying the denaturation of actin protein in the Ehrenberg’s Snapper mince fillets. Cumin and vitamin C treated mince fillets were found to go against the second thermodynamic in showing lower enthalpies of 3.10 and 37.7 J/g compared to 48.0 J/g for the reference. No correlation could be found between FT-IR analysis and DSC because in FT-IR, the vibrational modes responses originate from all types of proteins present in the mince fillets. DSC analysis is concerned with three types of proteins; myosin, sarcoplasmic and actin only.

## 4. Conclusions

The evidence from this study indicates that the presence of antioxidants helps in delaying the denaturation process of proteins. In general, FTIR gives an overall idea about the denaturation process, while DSC could assess changes in a specific protein; myosin, sarcoplasmic or actin. Taken together, these findings suggest that an increase in antioxidant contents in foods can rationally lead to a positive contribution to food quality and improve the protein nutrition quality. Nevertheless, more understanding is needed about the interaction of the antioxidant and the protein, as a result of the fact that natural source antioxidants and fish mince fillets are complex matrices. The prospect of being able to study interactions of a single polyphenol isolated from a specific source of antioxidant with a specific protein, serves as a continuous incentive to determine functionality in frozen mince fillets of Ehrenberg’s Snapper. The interaction of the antioxidant with the protein needs also to be explored at the cooking temperatures to have more understanding about the bioavailability of the proteins and antioxidants before joining the gastrointestinal tract.

## Figures and Tables

**Table 1:** Transition temperature, T_m_ (°C), and enthalpy ΔH^0^ (J/g) for antioxidant treated and non-treated minced fillets of Ehrenberg’s Snapper at −25.0 °C at storage time of 4 weeks.

Sample	Peak 1		Peak 2		Peak 3	
	T_m_ (°C)	ΔH^0^_U_ (J/g)	T_m_ (°C)	ΔH^0^_U_ (J/g)	T_m_ (°C)	ΔH^0^U (J/g)
Reference	47.0	5.95	61.9	48.0	108.6	200.9
Turmeric	-	-	75.5	73.7	112.0	482.8
Garlic	49.0	7.17	71.3	70.1	110.3	431.2
Cinnamon	41.0	769	-	-	105.8	239.0
Cumin	-	91.2	76.1	3.10	111.4	248.0
Ginger	-	-	-	-	102.0	1367
Vitamin C	48.0	28.7	65.0	37.7	107.6	340.5

## Supplementary Materials

The Supplementary Materials are available online at https://www.mdpi.com/2218-273X/9/9/442/s1.
