# Supplementary material for: The Effects of Storage on Quality and Nutritional Values of Ehrenberg’s Snapper Muscles (Lutjanus Ehrenbergi): Evaluation of Natural Antioxidants Effect on the Denaturation of Proteins
Source: Biomolecules. 2019 Sep 2;9(9):442. doi: 10.3390/biom9090442 (PMC6770199; doi:10.3390/biom9090442)
Supplement: Supplementary file 1 [file biomolecules-09-00442-s001.pdf]

Supplementary data – Data for TPC, flavonoids contents, DPPH elemental analysis of fish, FTIR and DSC of antioxidant treated and non-treated Ehrenberg snapper.

Table S1. Total phenolic content in mg (GAE)/100 g (DW) of garlic, ginger, cumin, turmeric, and cinnamon

Antioxidant	**mg(GAE)/100 DW**
Garlic	3.73 ± 0.01
Ginger	5.92 ±0.02
Cumin	13.26 ±0.01
Turmeric	20.34 ±0.01
Cinnamon	37.93 ± 0.19

Table S2. Estimated total flavonoid contents of different natural antioxidants as ppm of rutin

Antioxidant	**10. mg/mL**	**1.0 mg/mL**	**0.5 mg/mL**
Garlic	26.13 ± 0.01	1.80 ± 0.001	0.58 ± 0.31
Ginger	62.04 ± 0.02	4.28 ± 0.01	1.39 ± 0.07
Cumin	129.46 ± 0.05	8.93 ± 0.01	2.89 ± 0.15
Turmeric	70.85 ± 0.03	4.89 ± 0.01	1.58 ± 0.08
Cinnamon	44.51 ± 0.02	3.07 ± 0.001	0.99 ±0 .05

Table S3. DPPH scavenging activity (%) of ginger, cinnamon, cumin, turmeric, vitamin C and garlic

Antioxidant	**10. mg/mL**	**1.0 mg/mL**	**0.5 mg/mL**
Cinnamon	90.50 ±0.01	10.42 ± 0.06	3.39 ± 0.04
Fresh garlic	7.10 ± 0.01	0.83 ± 0.004	0.27 ± 0.01
Ginger	90.54 ± 0.01	10.42 ± 0.06	3.40 ± 0.04
Turmeric	80.45 ± 0.01	9.26 ± 0.05	3.01 ± 0.04
Cumin	89.87 ± 0.01	10.34 ± 0.06	3.37 ± 0.04
Vitamin C	26.26 ± 0.01	3.026 ± 0.02	0.98 ± 0.01

Table S4. Element analysis of Ehrenberg's Snapper ash

Element	Mass [%]	mg/Kg	Intensity[cps/mA]
^{12}Mg	1.42	1159.67	11.99
^{15}P	24.06	19649.00	2468.81
^{16}S	3.23	2637.83	403.94
^{17}Cl	2.38	1943.67	298.5
^{19}K	54.89	44826.83	6343.13
^{20}Ca	13.09	10690.17	953.49
^{26}Fe	0.31	253.17	99.28
^{30}Zn	0.21	171.50	153.62
^{38}Sr	0.41	334.83	583.24

Table S5. Amide I, Amide II and Amide A transmittance values of antioxidant treated and non-treated samples frozen at -25 oC for 1 week, 2weeks, 3 weeks and 4-weeks storage timing.

Date	Peak type	Reference		Cinnamon		Garlic		Ginger		Turmericin		Cumin		Vitamin C	
		$\nu(cm^{-1})$	Trans	$\nu(cm^{-1})$	Trans	$\nu(cm^{-1})$	Trans	$\nu(cm^{-1})$	Trans	$\nu(cm^{-1})$	Trans	$\nu(cm^{-1})$	Trans	$\nu(cm^{-1})$	Trans
Week-1	Amide A 3300 NH stretch	3299.944	0.50538	3299.944	0.46547	3299.944	0.57851	3299.944	0.46575	3299.944	0.59054	3299.944	0.48834	3299.944	0.48438
			0.51138		0.53147		0.65251		0.52275		0.62354		0.50334		0.51138
			0.51738		0.59747		0.72651		0.57975		0.65654		0.51834		0.53838
			±0.006		±0.066		±0.074		±0.057		±0.033		±0.015		±0.027
	Amide I 1600–1690 C=O stretch	1634.953	0.56936	1634.953	0.59969	1643.535	0.64828	1642.105	0.57338	1634.953	0.58004	1642.105	0.61003	1634.953	0.57136
			0.62236		0.62869		0.69028		0.62538		0.61904		0.62103		0.62236
			0.67536		0.65769		0.64828		0.67738		0.65804		0.63203		0.67336
			±0.053		±0.029		±0.042		±0.052		±0.039		±0.006		±0.051
	Amide II 1480–1575 CN stretch NH bending	1549.128	0.72931	1549.128	0.73186	1549.128	0.78289	1549.128	0.75324	1547.698	0.66201	1549.128	0.73206	1549.128	0.80731
			0.75731		0.76186		0.80089		0.75724		0.71101		0.75906		0.75731
			0.78531		0.79186		0.81889		0.76124		0.76001		0.78606		0.80731
			±0.028		±0.003		±0.018		±0.004		±0.049		±0.027		±0.005
Week-2	Amide A 3300 NH stretch	3299.943	0.47734	3299.944	0.49232	3299.944	0.48294	3299.94	0.47704	3299.944	0.5124	3299.944	0.50352	3299.944	0.48852
			0.52234		0.51032		0.50694		0.51104		0.5514		0.52152		0.51552
			0.56734		0.52832		0.53094		0.54504		0.5904		0.53952		0.54252
			±0.045		±0.018		±0.024		±0.034		±0.039		±0.018		±0.027
	Amide I 1600–1690 C=O stretch	1634.952	0.59334	1634.953	0.56349	1634.953	0.58094	1634.953	0.5283	1633.522	0.46895	1634.953	0.58063	1634.953	0.55223
			0.62934		0.60149		0.59794		0.5493		0.51295		0.61363		0.59223
			0.66534		0.63949		0.61494		0.5703		0.55695		0.64663		0.63223
			±0.036		±0.038		±0.017		±0.021		±0.044		±0.033		±0.040

	**Amide II 1480–1575 CN stretch, NH bending**	1549.128	0.69048	1549.128	0.682	1547.698	0.70426	1547.698	0.60891	1547.698	0.59117	1549.128	0.70683	1547.698	0.70802
			0.76548		0.732		0.72826		0.66191		0.60217		0.74283		0.71702
			0.84048		0.782		0.75226		0.71491		0.61317		0.77883		0.72602
			±0.075		±0.05		±0.024		±0.053		±0.011		±0.036		±0.009
**Week-3**	**Amide A 3300 NH stretching**	3299.943	0.49085	2999.55	0.78459	3299.944	0.43806	3299.944	0.49528	3299.944	0.4808	3301.374	0.45346	3299.944	0.47885
			0.50085		0.86159		0.50606		0.50628		0.5488		0.50846		0.50085
			0.51085		0.93859		0.57406		0.51728		0.6168		0.56346		0.52285
			±0.01		±0.077		±0.068		±0.011		±0.068		±0.055		±0.022
	**Amide I 1600–1690 C=O stretch**	1634.952	0.55516	1634.953	0.53463	1634.953	0.53885	1634.953	0.53669	1634.953	0.48196	1634.953	0.54212	1634.953	0.62116
			0.64616		0.61863		0.61585		0.62569		0.54496		0.63812		0.64616
			0.73716		0.70263		0.69285		0.71469		0.60796		0.73412		0.67116
			±0.091		±0.084		±0.077		±0.089		±0.063		±0.096		±0.022
	**Amide II 1480–1575 CN stretch NH bending**	1634.953	0.60716	1549.128	0.67242	1549.128	0.60229	1549.128	0.68625	1547.698	0.52599	1549.128	0.73035	1550.559	0.74912
			0.64616		0.75642		0.75529		0.76725		0.64199		0.78135		0.79512
			0.68516		0.84042		0.90829		0.84825		0.65359		0.83235		0.84112
			±0.039		±0.084		±0.153		±0.081		±0.0116		±0.051		±0.046
**Week-4**	**Amide A 3300 NH stretch**	2999.558	0.8337	3299.944	0.45725	3299.944	0.41108	3299.944	0.45225	2999.559	0.78016	3299.944	0.46918	2999.559	0.77868
			0.8727		0.51725		0.50808		0.50325		0.85116		0.50418		0.86068
			0.9117		0.57725		0.60508		0.55425		0.92216		0.53918		0.94168
			±0.039		±0.060		±0.097		±0.051		±0.071		±0.035		±0.081
	**Amide I 1600–1690 C=O stretch**	1634.953	0.65331	1634.953	0.66455	1634.953	0.61647	1634.953	0.65523	1633.52	0.49238	1634.953	0.64657	1633.522	0.58776
			0.67031		0.67955		0.64747		0.66223		0.51338		0.66357		0.61976
			0.68731		0.69455		0.67847		0.66923		0.53438		0.68057		0.65176
			±0.017		±0.022		±0.022		±0.007		±0.021		±0.017		±0.032
	**Amide II 1480–1575 CN stretch, NH bending**	1549.128	0.79449	1574.876	0.75328	1549.128	0.68594	1550.559	0.74015	1550.559	0.7023	1550.559	0.74433	1549.128	0.30511
			0.80549		0.83328		0.79594		0.81715		0.6063		0.81833		0.75511
			0.81649		0.91328		0.90594		0.89415		0.7023		0.89233		0.80011
			±0.011		±0.080		±0.11		±0.077		±0.096		±0.074		±0.045

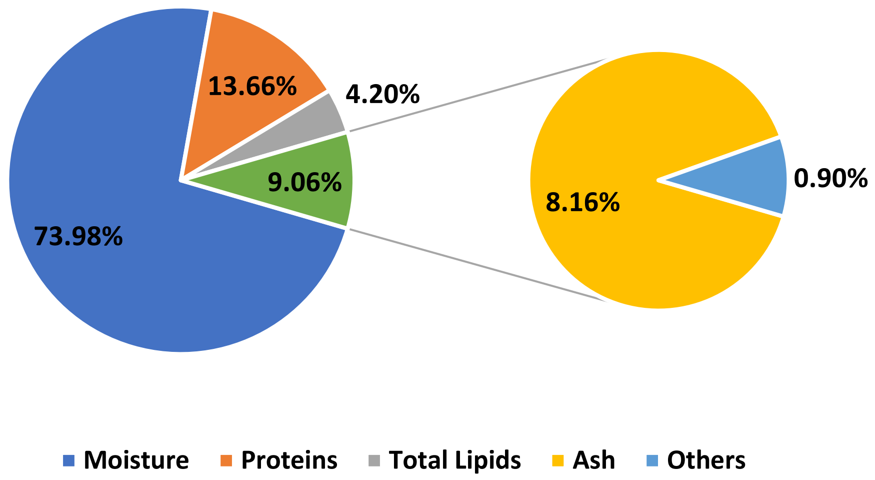

Figure S1 composition of Ehrenberg's Snapper black spot fish

1. FT-IR spectra

FT-IR spectra of treated and non-treated Ehrenberg's snapper are given in Figures 2S to 5S.

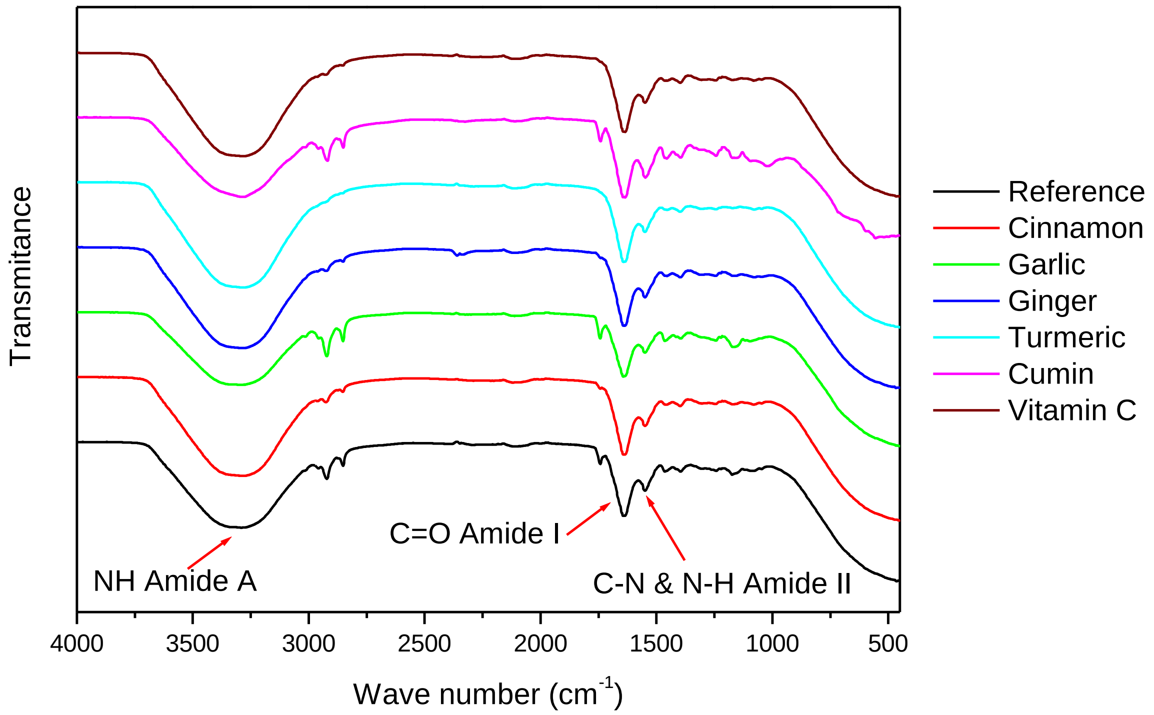

Figure. S2 FTIR for frozen fish (-25.0˚C) Ehrenberg's Snapper treated with: 150mg garlic; 150mg cinnamon; 150mg cumin; 150mg turmeric; 150mg garlic; 150mg ginger; and 500 ppm vitamin C; and without antioxidant for control after **1-week** time.

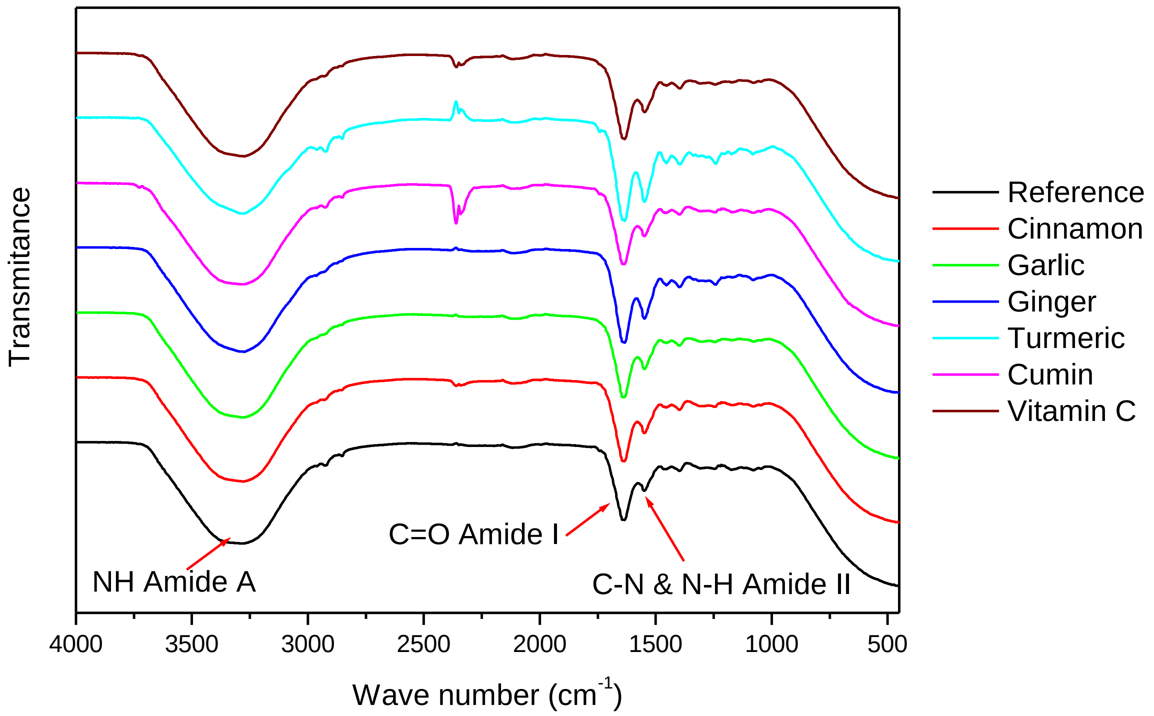

Figure. S3 FTIR for frozen fish (-25.0˚C) Ehrenberg's Snapper treated with: 150mg garlic; 150mg cinnamon; 150mg cumin; 150mg turmeric; 150mg garlic; 150mg ginger; and 500 ppm vitamin C; and without antioxidant for control after **2-week** time.

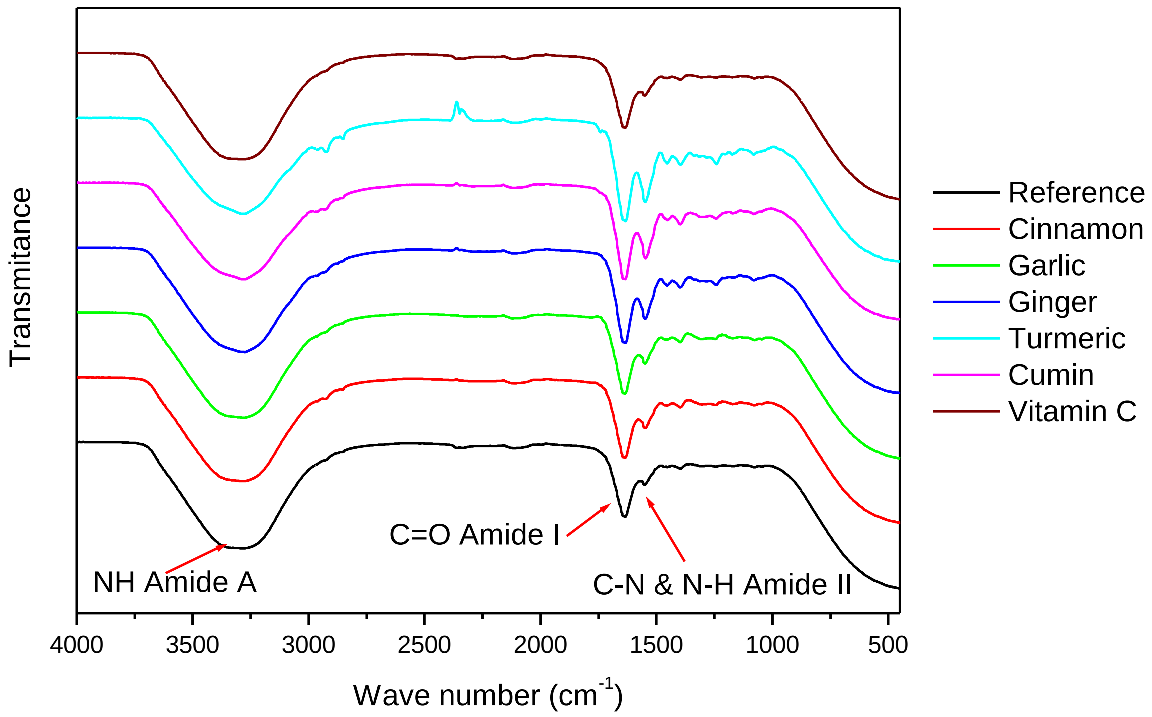

Figure. S4 FTIR for frozen fish (-25.0˚C) Ehrenberg's Snapper treated with: 150mg garlic; 150mg cinnamon; 150mg cumin; 150mg turmeric; 150mg garlic; 150mg ginger; and 500 ppm vitamin C; and without antioxidant for control after **3-week** time.

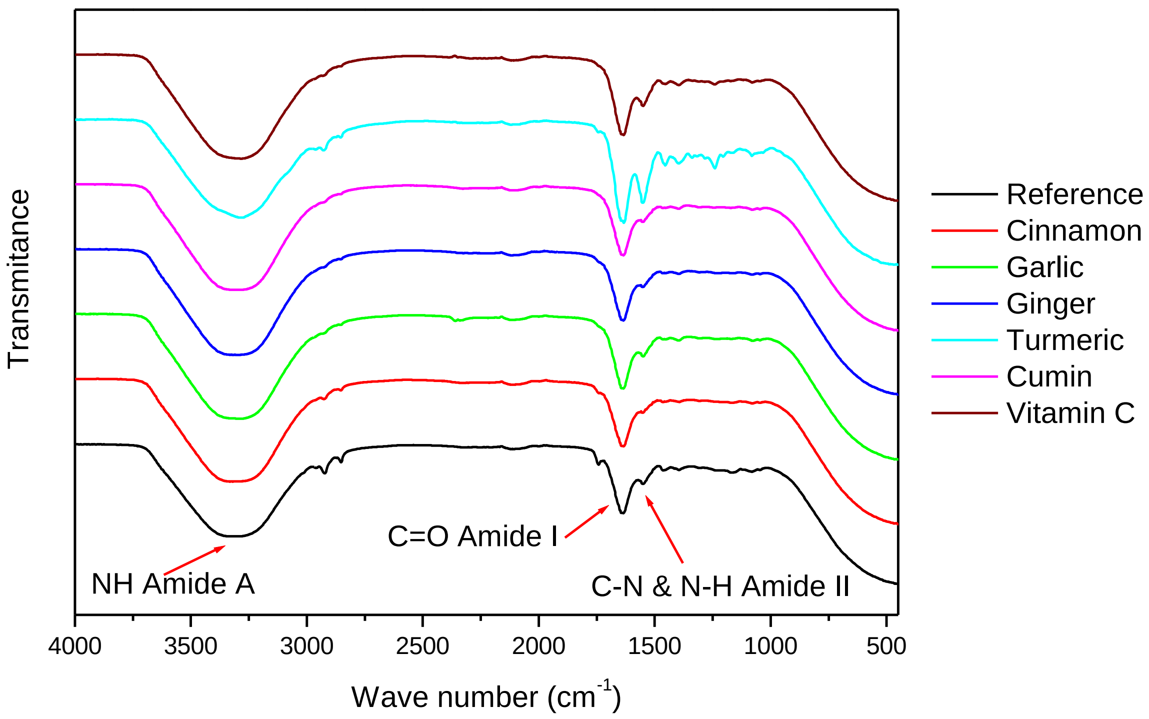

Figure. S5 FTIR for frozen fish (-25.0˚C) Ehrenberg's Snapper treated with: 150mg garlic; 150mg cinnamon; 150mg cumin; 150mg turmeric; 150mg garlic; 150mg ginger; and 500 ppm vitamin C; and without antioxidant for control after **4-week** time.

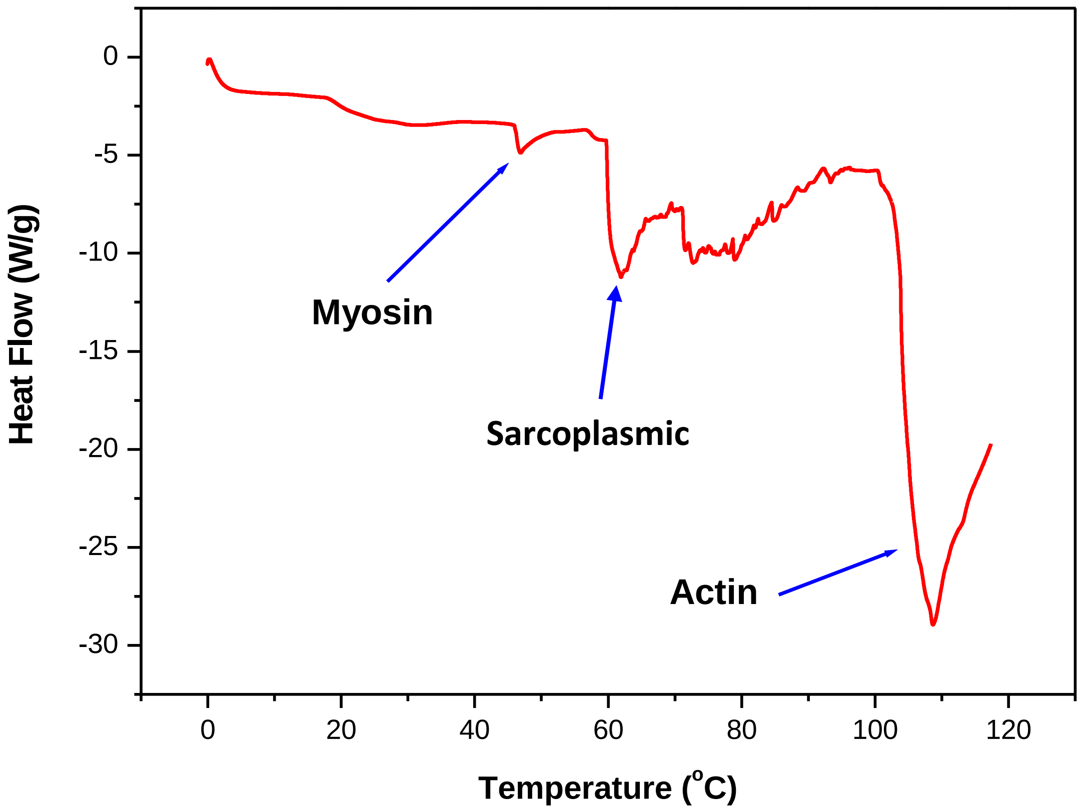

Figure S6. Differential scanning calorimetry of Ehrenberg snapper during storage of 30 days at -25˚C without antioxidant for control.

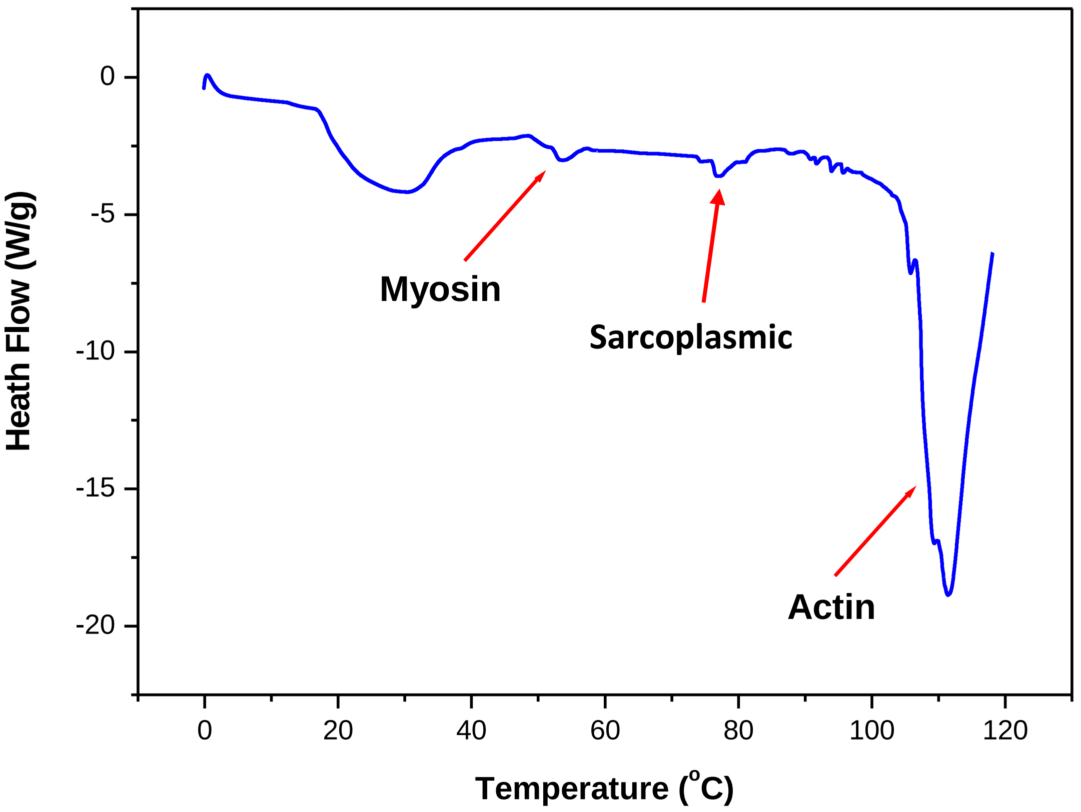

Figure S7. Differential scanning calorimetry of Ehrenberg snapper during storage of 30 days at -25˚C with: 150mg cumin.

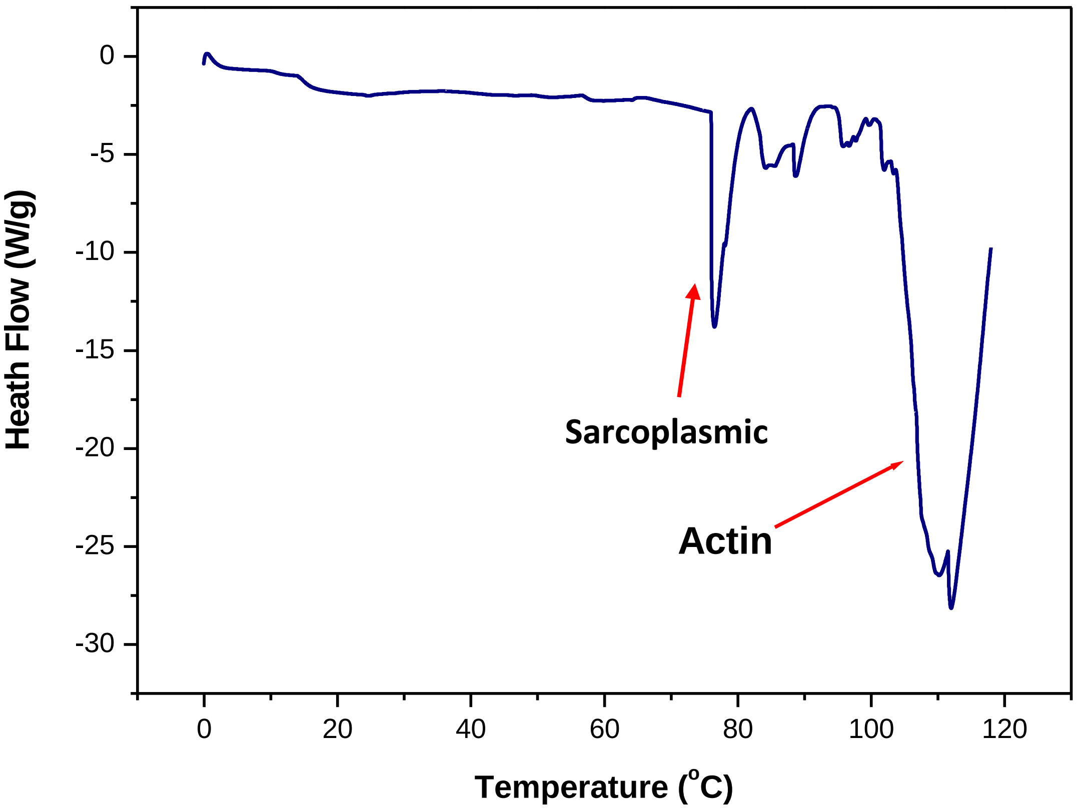

Figure S8. Differential scanning calorimetry of Ehrenberg snapper during storage of 30 days at -25˚C with: 150mg turmeric.

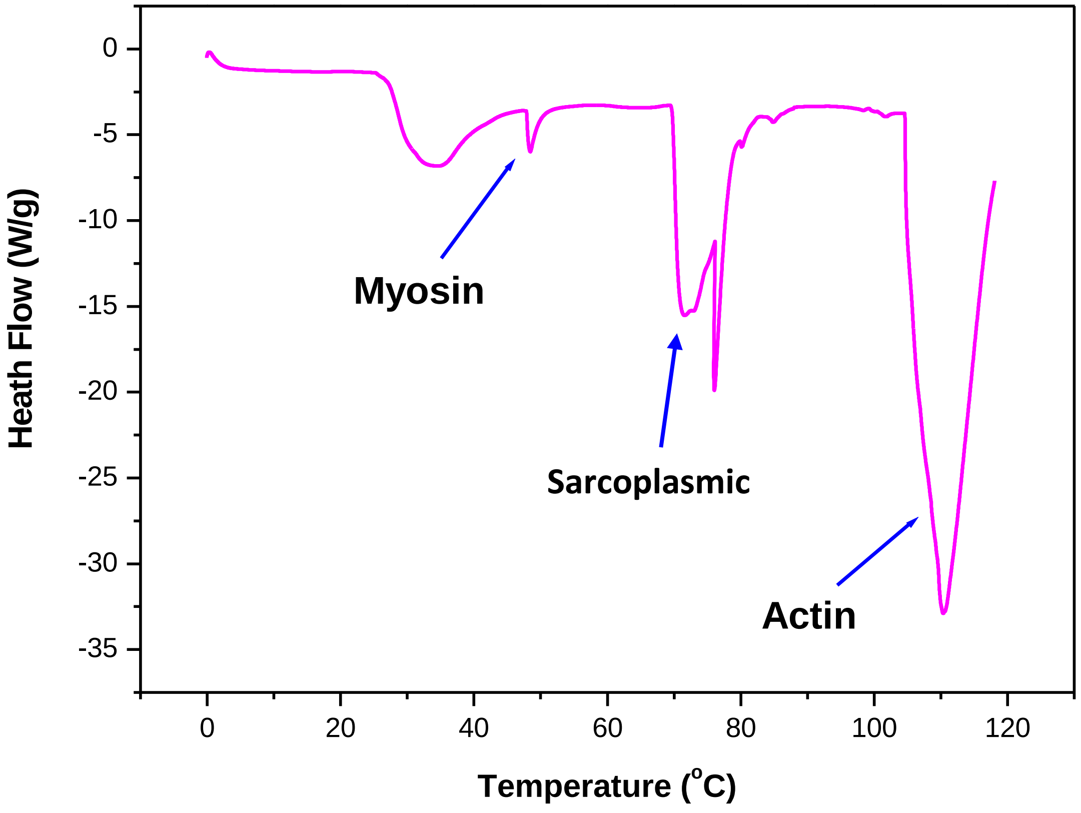

Figure S9. Differential scanning calorimetry of Ehrenberg snapper during storage of 30 days at -25˚C with: 150mg garlic.

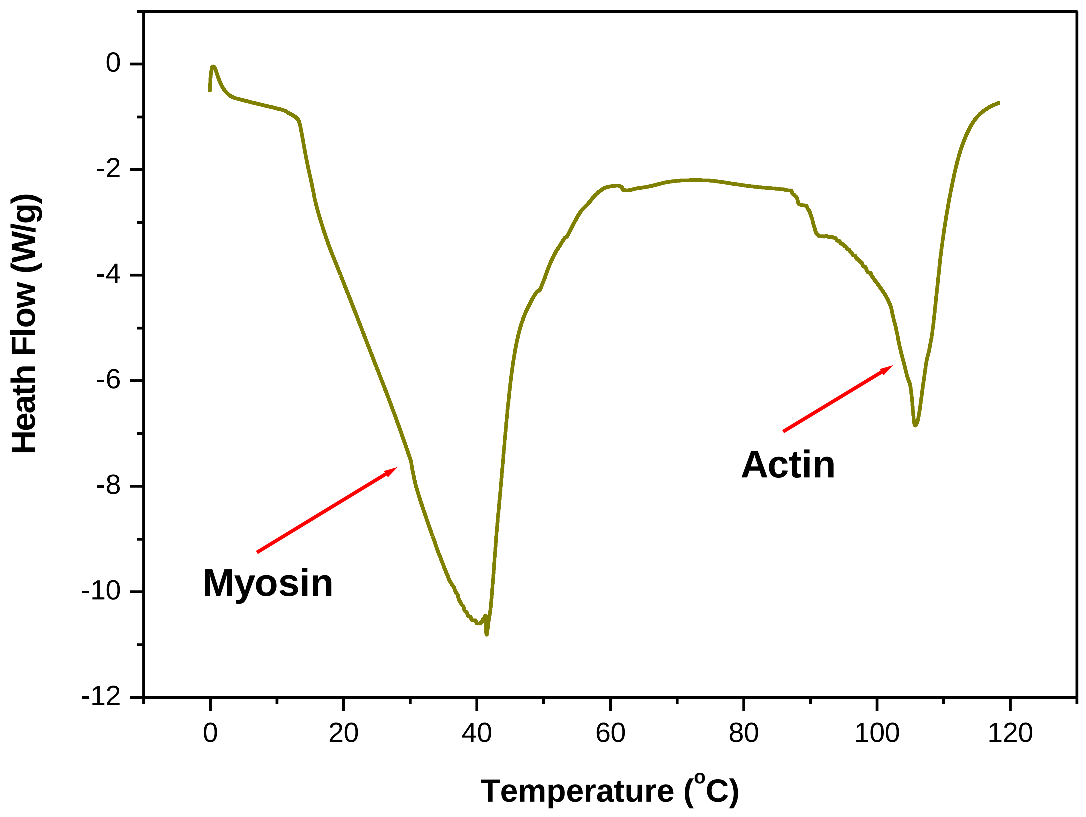

Figure S10. Differential scanning calorimetry of Ehrenberg snapper during storage of 30 days at -25˚C with: 150mg cinnamon.

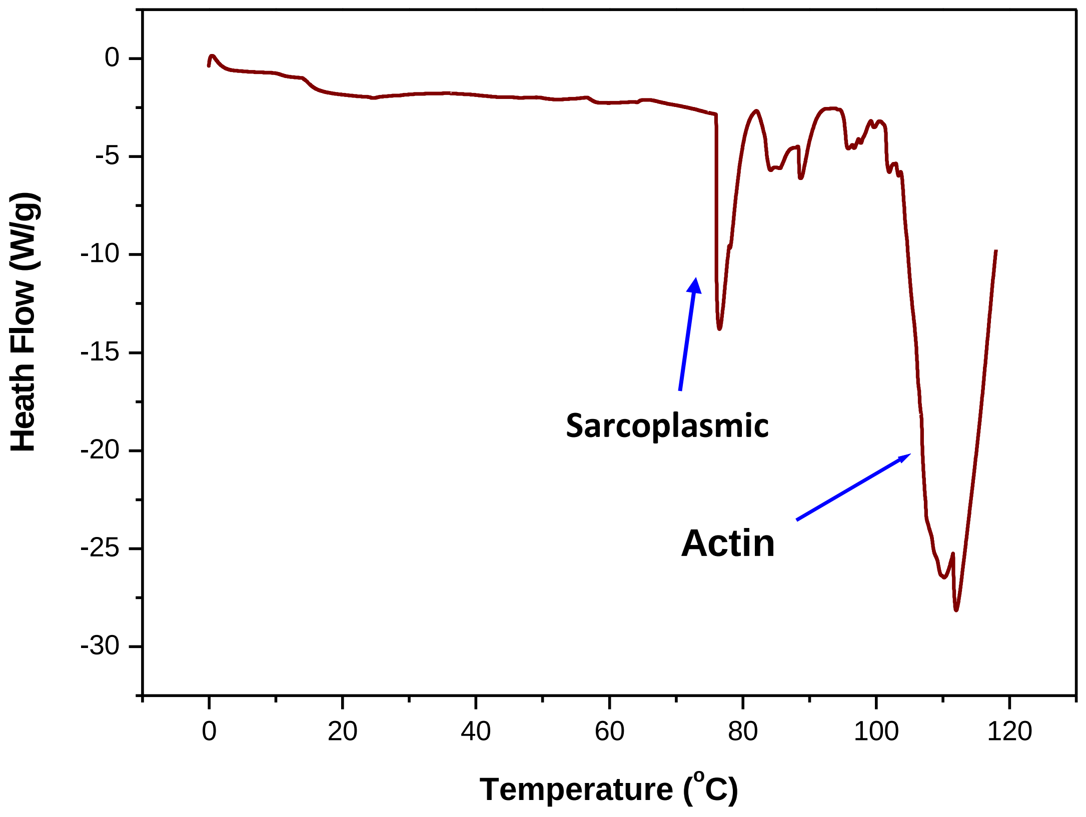

Figure S11. Differential scanning calorimetry of Ehrenberg snapper during storage of 30 days at -25˚C with: 150mg ginger.

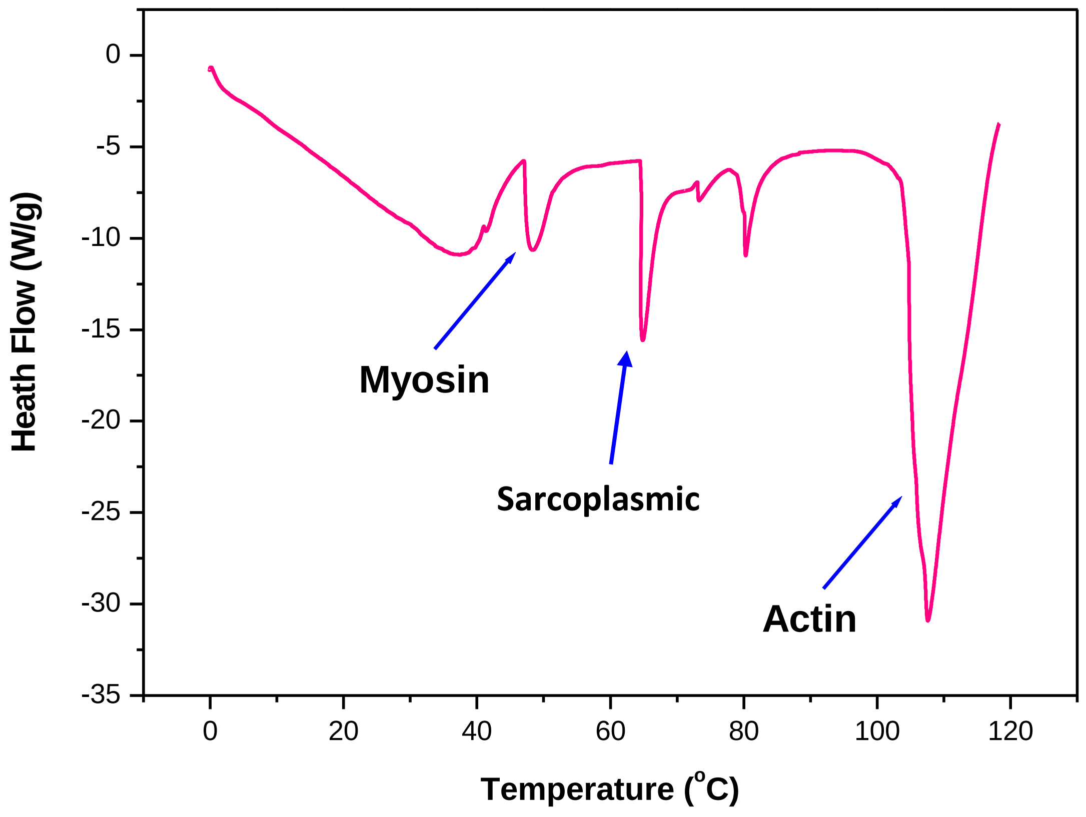

Figure S12. Differential scanning calorimetry of Ehrenberg snapper during storage of 30 days at -25˚C with: 500 ppm vitamin C.
